# Pneumatic Displacement with Perfluoropropane Gas and Intravitreal Tissue Plasminogen Activator for Subretinal Subfoveal Hemorrhage after Focal Laser Photocoagulation in Central Serous Chorioretinopathy

**DOI:** 10.1155/2014/592746

**Published:** 2014-11-17

**Authors:** Khalid Al Rubaie, Juan V. Espinoza, Andres F. Lasave, Dario Savino-Zari, Fernando A. Arevalo, J. Fernando Arevalo

**Affiliations:** ^1^King Khaled Eye Specialist Hospital, Al-Oruba Street, P.O. Box 7191, Riyadh 11462, Saudi Arabia; ^2^The Retina & Vitreous Service, Clínica Oftalmológica Centro Caracas, Avenida Panteon, Caracas 1010, Venezuela; ^3^Instituto de Otorrinolaringología, Avenida Cajigal, Caracas 1010, Venezuela; ^4^The Retina Division, Wilmer Eye Institute, Johns Hopkins University School of Medicine, 1300 Thames Street, Baltimore, MD 21231, USA

## Abstract

*Objective*. To report the visual and anatomic outcomes of pneumatic displacement with perfluoropropane (C3F8) gas and intravitreal tissue plasminogen activator (IVTPA) for subretinal subfoveal hemorrhage after focal laser photocoagulation in central serous chorioretinopathy (CSCR).* Method*. Interventional, retrospective case report of one eye (one patient). Outcome measures included visual acuity (VA), central macular thickness (CMT), and size of the lesion at two weeks of followup. Fluorescein angiography (FA) and optical coherent tomography (OCT) were used to measure anatomic outcomes.* Results*. A 35-year-old man with history of chronic CSCR received focal laser photocoagulation in the right eye two days before presentation. At initial examination, VA was 20/200 (ETDRS chart), CMT was 398 *μ*, and a subretinal subfoveal hemorrhage was seen. Tissue plasminogen activator (tPA) at a dose of 25 *µ*g/0.1 mL was injected intravitreally before intravitreal C3F8 injection, and prone positioning was indicated postoperatively. At 24 hours, the hemorrhage had been displaced inferiorly and VA improved to 20/100. Two weeks later, VA improved to 20/80, CMT decreased to 225 *μ*, and the hemorrhage decreased without foveal involvement.* Conclusions*. The technique seems safe and effective in treating visually significant subretinal subfoveal hemorrhage.

## 1. Introduction

Central serous chorioretinopathy (CSCR) is an idiopathic disorder of the outer blood-retinal barrier, defined by the presence of serous sensory retinal detachment (RD) with an active retinal pigment epithelium (RPE) leakage without any evidence of other ocular or systemic disorders known to produce a similar presentation. The condition is seen predominantly in males with a ratio of 10 : 1. In most cases, CSCR is self-limited and resolves spontaneously in 4 to 6 months; however, when it does not resolve during this time frame the condition is then called chronic CSCR.

Management options for CSCR include observation, photodynamic therapy, intravitreal antivascular endothelial growth factors (anti-VEGF), or thermal laser photocoagulation [1–6]. While selected cases of acute CSCR benefit from retinal (thermal) laser photocoagulation, there is no standard treatment for chronic CSCR [4]. Complications related to laser treatment include accidental photocoagulation of the fovea, foveal distortion, scotomas, significant loss of contrast sensitivity, a 2% to 10% risk of developing choroidal neovascularization within several weeks to months after treatment, and subretinal hemorrhage [1, 5].

Experimental studies have demonstrated that irreversible retinal damage occurs as early as 24 hours after the onset of subretinal hemorrhage [7–9]. Interestingly some studies have indicated that the use of the fibrinolytic agent recombinant tissue plasminogen activator (tPA) combined with intravitreal gas may have a beneficial effect over massive subretinal hemorrhage in age macular degeneration (AMD), ruptured macroaneurysm, and trauma [7, 10–12].

The purpose of this case is to report the visual and anatomic outcomes of pneumatic displacement with perfluoropropane (C3F8) gas and intravitreal tissue plasminogen activator (IVTPA) for subretinal subfoveal hemorrhage after focal laser photocoagulation in CSCR.

## 2. Case Report

**Figure 1:**
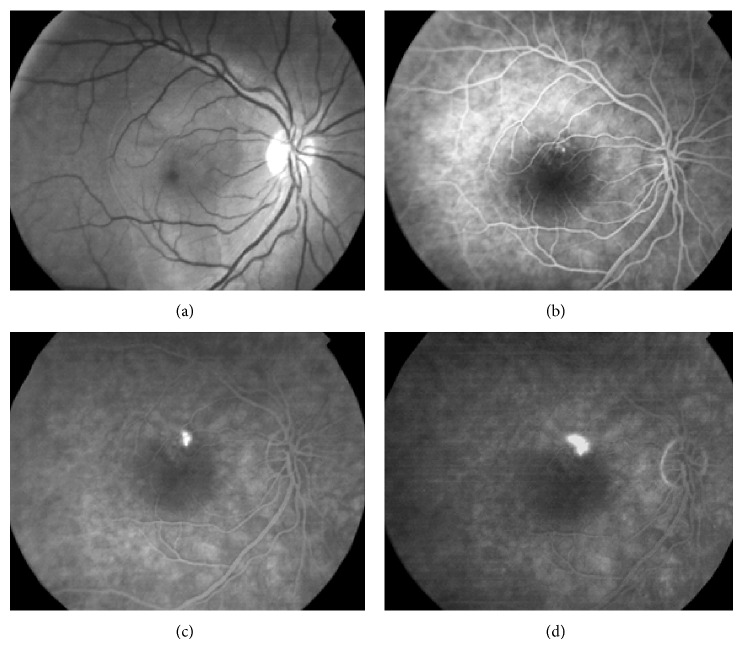
(a) Red-free photograph shows the right eye before thermal laser therapy with a large macular serous retinal detachment measuring about 4 disc diameters. (b) Early fluorescein angiography (FA) phase shows a small pinpoint area of retinal pigment epithelium (RPE) leak superonasal to the fovea. (c) Midphase fluorescein angiography (FA) shows an increase in the small focal area of RPE leak superonasal to the fovea. (d) Late phase FA shows an increase in the superonasal focal hyperfluorescence with an inkblot-like leakage pattern.

**Figure 2:**
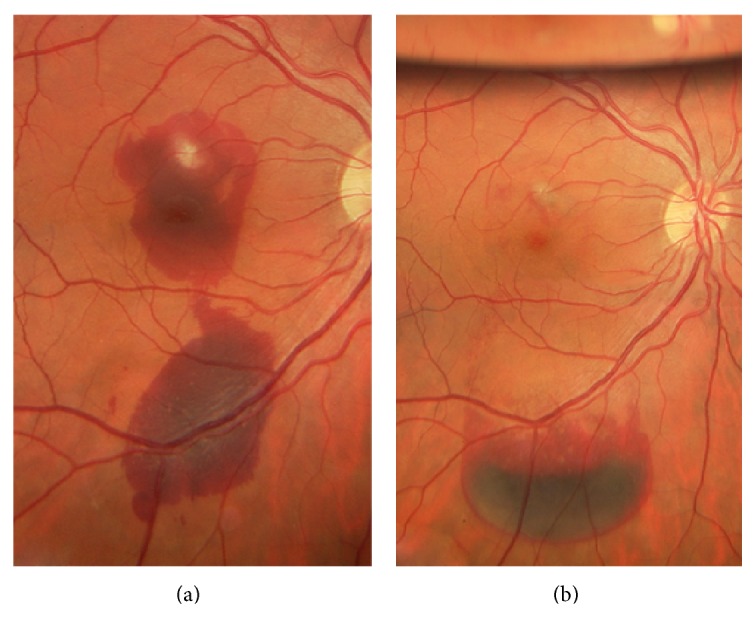
(a) Color photograph of the right eye two days after focal thermal laser. At initial examination, visual acuity (VA) was 20/200 (ETDRS chart) and a subretinal subfoveal hemorrhage with the greatest linear diameter (GLD) of 4.500 *μ* was seen. (b) Color photograph after pneumatic displacement with intravitreal tissue plasminogen activator (IVTPA) combined with pure C3F8. At 24 hours, the hemorrhage had been displaced inferiorly and VA improved to 20/100. Two weeks after pneumatic displacement with C3F8 and IVTPA, VA improved to 20/80.

**Figure 3:**
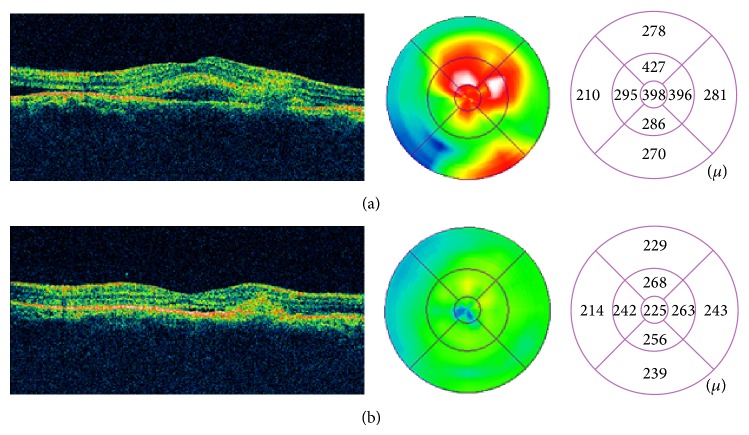
(a) Optical coherence tomography (OCT) revealed an increased central macular thickness (CMT) of 398 *μ* with loss of foveal architecture and subretinal hemorrhage with a neurosensory retinal detachment. (b) Two weeks after pneumatic displacement with C3F8 and intravitreal tissue plasminogen activator, visual acuity improved to 20/80 and CMT decreased to 225 *μ*. A hyperreflective fusiform elevation of the retinal pigment epithelium/choriocapillaris complex that seems to correspond to fibrosis tissue is seen at the thermal laser site. A small subfoveal neurosensory retinal detachment persists. However, there is normalization of the foveal architecture.

A 35-year-old man with history of chronic CSCR received focal laser photocoagulation in the right eye (RE) two days before presentation. Argon laser has been applied directly to the fluorescein angiographic (FA) leakage site in the RE (Figure 1). According to the referring physician, laser parameters included a spot size of 200 *μ*m for 100 ms at a power of 100 to 200 mw with 6 spots. Immediately after treatment he suffered a marked decrease of VA of the RE. At initial examination, VA was 20/200 (ETDRS chart) and a subretinal subfoveal hemorrhage with the greatest linear diameter (GLD) of 4.500 *μ* was seen (Figure 2(a)). Optical coherence tomography (OCT) revealed an increased central macular thickness (CMT) of 398 *μ* with loss of foveal architecture and subretinal hemorrhage with a neurosensory retinal detachment (Figure 3(a)). Under topical and subconjunctival anesthesia, tissue plasminogen activator (tPA) at a dose of 25 *μ*g/0.1 mL was injected intravitreally 40 minutes before intravitreal 0.3 mL of pure C3F8 injection with a previous paracentesis, and prone positioning was indicated postoperatively. At 24 hours, the hemorrhage had been displaced inferiorly and VA improved to 20/100. Intermittent face down position was continued for two weeks. Two weeks after pneumatic displacement with C3F8 and IVTPA, VA improved to 20/80, CMT decreased to 225 *μ*, and GLD of the hemorrhage decreased to 2000 *μ* without foveal involvement (Figures 2(b) and 3(b)). In addition, OCT demonstrates a hyperreflective fusiform elevation of the RPE/choriocapillaris complex that seems to correspond to fibrosis tissue at the thermal laser site. A small subfoveal neurosensory retinal detachment persists. However, there is normalization of the foveal architecture (Figure 3(b)).

## 3. Comment

Laser treatment for the early management of exudative manifestations in CSCR remains a controversial issue, particularly because most cases of CSCR have spontaneous resolution after a period of observation with favorable visual outcomes. A possible mechanism for laser treatment is debridement of diseased RPE permitting ingrowth of surrounding healthy RPE and resolution of CSCR [1]. In this case report the patient suffered a massive subretinal hemorrhage after thermal laser application causing a devastating anatomic and functional complication. It may be caused by a rupture of Bruch's membrane.

Intravitreal injection of tPA and gas followed by prone positioning is effective in displacing thick subretinal hemorrhage [7, 10–12]. The procedure is technically simple, and the rate of serious complications is low compared with surgical subretinal clot extraction by pars plana vitrectomy. Rapid removal of blood from the macula is important to recover the patient's vision and to minimize the adverse effects of clot retraction, iron toxicity, and the metabolic barrier of the blood clot [10].

In summary, this technique seems to be safe and effective for treating massive subretinal subfoveal hemorrhage. However, visual recovery is limited by the underlying macular pathology. Focal laser photocoagulation for the treatment of CSCR may cause a rupture of Bruch's membrane and a subretinal subfoveal hemorrhage. Alternative therapies to laser photocoagulation including photodynamic therapy, intravitreal antivascular endothelial growth factors, or a combination of both need to be considered [1, 3–13].
